# Supplementary material for: Biomimetic Liquid Crystal-Modified Mesoporous Silica−Based Composite Hydrogel for Soft Tissue Repair
Source: J Funct Biomater. 2023 Jun 8;14(6):316. doi: 10.3390/jfb14060316 (PMC10299385; doi:10.3390/jfb14060316)
Supplement: Supplementary file 1 [file jfb-14-00316-s001.zip › jfb-2373532-supplementary.pdf]

Supplementary Material

Biomimetic Liquid Crystal-Modified Mesoporous Silica−Based Composite Hydrogel for Soft Tissue Repair

Xiaoling Li [1,†], Lei Wan [1,†], Taifu Zhu [1], Ruiqi Li [1], Mu Zhang [1] and Haibin Lu [1,2,*]

S1. Experimental method

S1.1. Sythesis of the LC

First, the undecylcholesteryl ester monomer LC is prepared by a simple esterification reaction, followed by hydrosilylation to obtain the side chain polymer LC. The specific experimental method is as follows. Cholesterol (0.02 mol) was dissolved in 100 mL of dichloromethane (DCM), and 4-dimethylaminopyridine (DMAP, 0.0012 mol) and undecylenic acid (0.03 mol) was added to the above solution while stirring; finally, the dicyclohexylcarbodiimide (DCC, 0.03 mol) was added. The reaction was stirred at 30°C for 20 h in the dark, and the urea formed by the absorption of DCC was removed by filtration. The filtrate was washed successively with distilled water, NaOH solution, NaHCO3 solution, and distilled water and dried over anhydrous magnesium sulfate overnight. After filtration, the DCM solvent was evaporated under normal pressure to obtain a pale-yellow material. The pure cholestearyl undecanoate LC was obtained by recrystallization from ethanol.

S1.2. Biomimetic mineralization of CS/LC-MSN

The resulting CS/LC-MSN hydrogel scaffold was incubated in 50 mL of 1.5 SBF, where it was maintained at 37 °C for 2-6 days for mineral growth. The 1.5 SBF solution was mixed with Ca^{2+} (3.75 mM), Cl^- (150.0 mM), Na^+ (143.5 mM), HPO_4^{2-} (1.5 mM), K^+ (6.0 mM), Mg^{2+} (1.5 mM), SO_4^{2-} (0.74 mM), and HCO_3^- (4.2 mM). The obtained biomineralized composite underwent successive cleaning and freeze-drying for the next measurement.

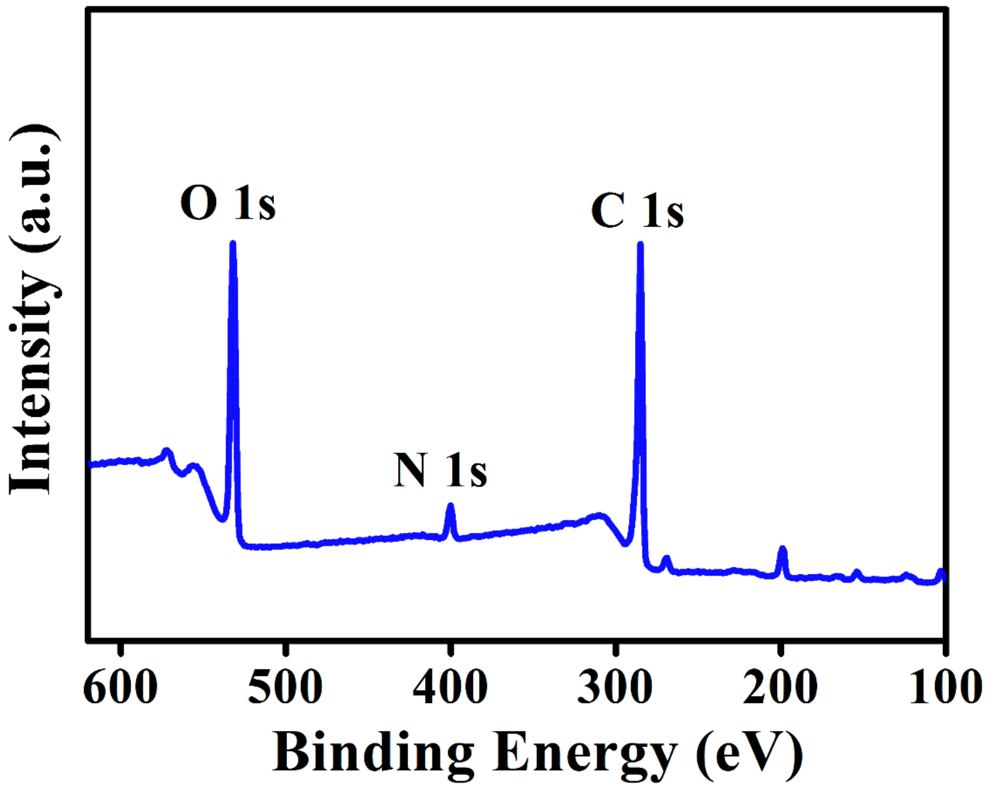

Figure S1. XPS survey spectrum of CS/LC-MSN composite hydrogels.

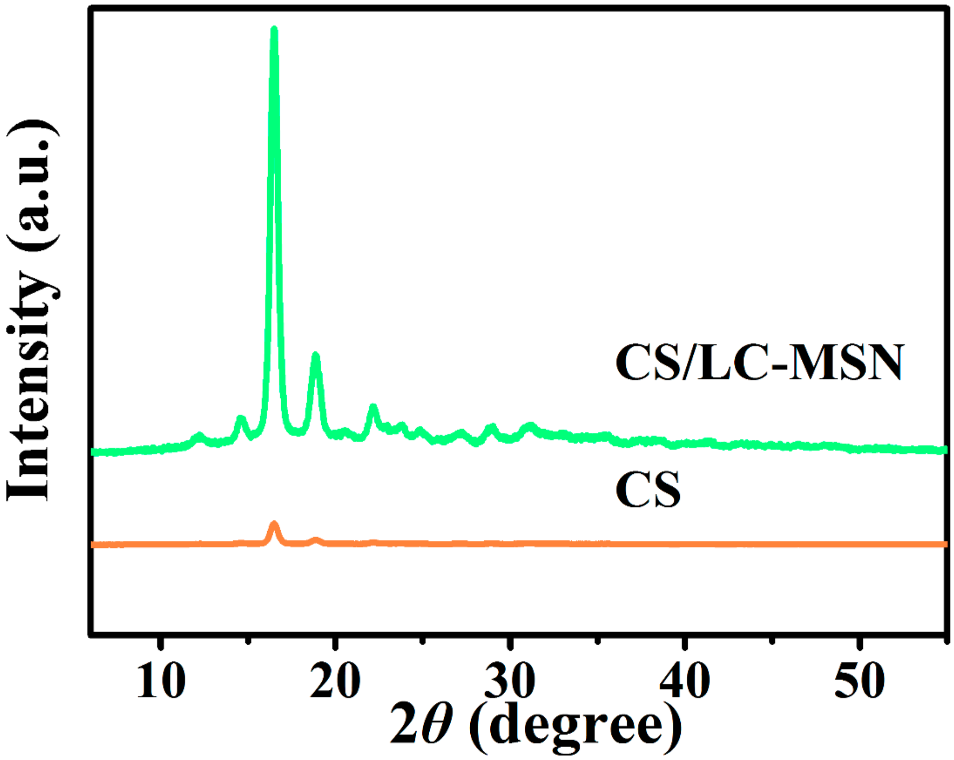

Figure S2. XRD pattern of CS and CS/LC-MSN specimens. The diffraction peaks of LC-MSN around 16.7 and 19.3 were observed, which corresponded to the o (200)/ (110) and (203) planes of crystalline, respectively.

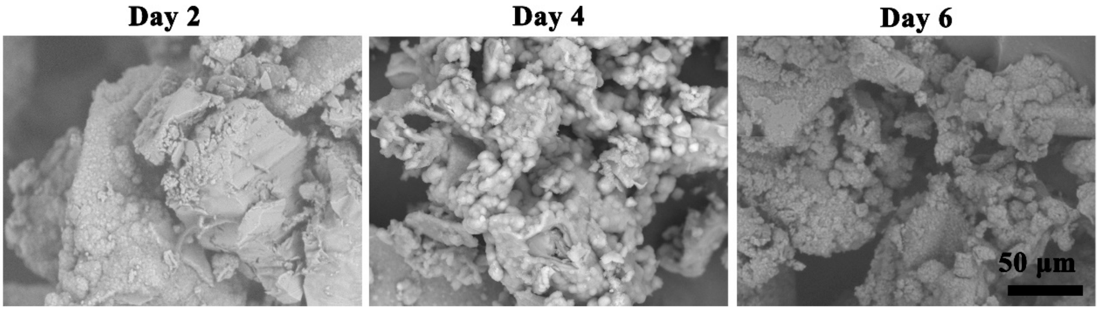

Figure S3. Morphologies of CS/LC-MSN composite hydrogel scaffolds after 2, 4, and 6 days of biomimetic mineralization.

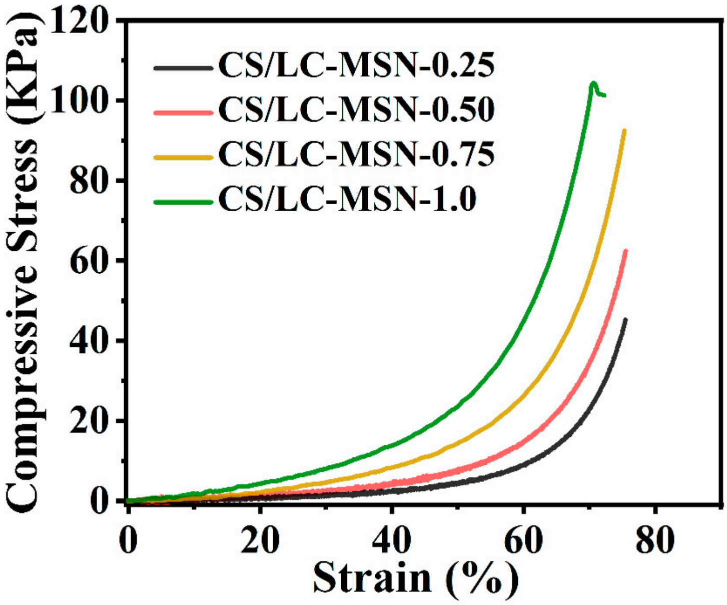

Figure S4. The compressive test of CS/LC-MSN composite hydrogels.
